# Swimming at the Time of COVID-19: A Cross-Sectional Study among Young Italian Competitive Athletes

**DOI:** 10.3390/ijerph192013236

**Published:** 2022-10-14

**Authors:** Francesca Gallè, Carmela Protano, Matteo Zaccarin, Stefano Zanni, Federica Valeriani, Giorgio Liguori, Vincenzo Romano Spica, Matteo Vitali

**Affiliations:** 1 Department of Movement Sciences and Wellbeing, University of Naples “Parthenope”, 80133 Naples, Italy; 2 Department of Public Health and Infectious Diseases, Sapienza University of Rome, 00185 Rome, Italy; 3 Department of Movement, Human and Health Sciences, University of Rome “Foro Italico”, 00135 Rome, Italy

**Keywords:** COVID-19, competitive athletes, infection control, sport, swimming pools

## Abstract

During the coronavirus disease 2019 (COVID-19) pandemic, several restriction measures were imposed to control the virus transmission, with important repercussions on different sectors, including sport. This study aimed to explore the effects of the COVID-19 pandemic on Italian competitive swimmers by analyzing how the disease and the restriction measures affected their training. In total, 396 competitive swimmers (mean age 16.0 ± 3.2 years) participated. A questionnaire was used to collect their general information, to assess whether they had had COVID-19 and the number of training days lost due to the disease or to the closure of swimming facilities, and the possible alternative training adopted. Twenty-four (6.1%) participants had had COVID-19 and lost, on average, 32 training days. The closure of facilities caused an interruption in swimming training for about 18% of the participants. The majority of these continued their training, mainly through home-based exercise, but reduced their weekly training time (-8 median hours/week). A positive association was found between regularly adopted weekly training volume and that assumed during pandemic closure (OR 9.433, CI95% 1.644–54.137, *p* = 0.012), suggesting that the previous level of engagement in sport can represent a predictor of exercise maintenance in challenging situations such as a pandemic. Further studies are needed to identify personal, environmental, and social resources that can help individuals to counteract the negative effects of restriction measures.

## 1. Introduction

Since its beginning, the coronavirus disease 2019 (COVID-19) pandemic has had negative consequences on people’s daily life and activities besides its direct health effects [1,2]. In order to control the virus transmission, several restriction measures were imposed worldwide, and also in Italy, with important social and economic repercussions in different settings [3,4]. The sport setting was also affected by these measures [5]. In the first phases of the pandemic, when the complete lockdown was adopted in many countries around the world, major sporting events were canceled or postponed and sport facilities were also inaccessible to athletes with the only exception of elite athletes [3,4,5,6,7,8,9]. In the subsequent phases, when the sport facilities were gradually reopened to the public, a lot of control measures such as shifts and distancing based on the acquired epidemiological knowledge were taken [5,9,10,11]. In this context, swimmers had to face not only an obliged stop of their training activities, but also one or more home confinement periods. Like other athletes, they had to exercise at home in order to maintain their fitness level and avoid detraining, defined as the loss of training-induced adaptations, with negative consequences on cardiovascular and muscle function, energy metabolism, and swimming speed and power [12,13]. However, considering the differences that exist between watersports and land-based sports, swimmers had to find new strategies to adapt their training to completely different conditions [14]. Training in a dry environment cannot totally replace exercising in a pool [15]. Home confinement and social restriction also had important psychological consequences on many athletes, such as an increase in psychological stress, which could have hindered their adaptive response to the changed life conditions [16].

This study aimed to explore the effects of the COVID-19 pandemic on Italian competitive swimmers by analyzing how the restriction measures and the disease affected their training volume, and to highlight the possible factors associated with their training during swimming pool closures.

## 2. Materials and Methods

### 2.1. Study Design and Population

This study is part of the cross-sectional study on Swimming Pools Health Related Aspects—SPHeRA—performed in 2021 on a population of young Italian competitive swimmers. A sample size of at least 385 swimmers was estimated with an expected prevalence (*p*) of 0.5, a margin of error (ε) of 0.05, and a confidence level set at 95%. In order to recruit the participants, we contacted each team by email with at least one swimmer registered for the championship, explained the research and how to participate, and invited all of the eligible swimmers to take part in the study. The study was performed according to the principles of the Declaration of Helsinki. Ethical approval was obtained from the Research Committee of the University of Rome “Foro Italico” (approval number CAR 92/2021).

### 2.2. Questionnaire

The questionnaire was created using other previously validated questionnaires, adapting the questions to suit our specific population and intent [17,18]. The questionnaire was subjected to a validation process ad hoc. First of all, some opinions were collected both from experts in the sport of swimming and from epidemiologists. A pilot test was then carried out and the questionnaire was filled in by 86 swimmers who met the selection criteria for the study to test the comprehensibility of the questions and their relevance with respect to the study population, the consistency between questions, the possible “floor effect” or “ceiling effect”, and the presence of any type of errors (both in content and form). At the end of the process, necessary changes were made to some questions. In addition, the data collected were used to calculate the Cronbach’s alpha for assessing the reliability of the test items. The Cronbach’s alpha was >0.80 for all of the investigated areas. The collection of responses took place at the end of two different national events of the 2020/2021 competitive calendar: the first following the conclusion of the winter–spring season and the second at the end of the summer season. The questionnaire was aimed to collect general information about each athlete such as age, gender, geographic area, and years of practice. We asked the athletes to report how many days of training they had lost due to the closure of swimming facilities to contain the COVID-19 pandemic. In addition, we investigated whether the athletes took part in another type of training during the closures due to the pandemic and in which place they trained. Finally, the athletes were asked to report whether they had been diagnosed with COVID-19 during their last season and how many training days they consequently lost.

### 2.3. Statistical Analysis

The data analysis was performed on the statistical software STATA^®^ (STATA 17.0, StataCorp LLC, College Station, TX, USA). At first, descriptive analysis was carried out; the arithmetic mean, median, and standard deviation were calculated for each continuous variable and the Shapiro–Wilk test was used to assess the normality. The weekly training volume was calculated by multiplying the number of hours per session with the number of sessions per week. The categorical variables were compared by chi-squared test (χ^2^), while the comparison between the continuous variables was performed using the Student’s *t* test for independent samples. In order to highlight the possible differences between those athletes who interrupted their training or exercised less and those who continued their training at a high level even during the closure of swimming pools, the median value of the autonomous weekly training volume assumed in this period was used as the cut-off. The variables related to the demographic characteristics and swimming practice of the two groups were compared through the χ^2^ test in order to identify those variables that showed significant differences. These variables were then included in a logistic regression analysis, considering the autonomous weekly training volume as the outcome (training volume ≤ median value = 0, >median value = 1).

## 3. Results

In total, 396 athletes took part in the study. Table 1 reports the characteristics of the study population, days of training lost, and alternative training declared by participants.

The sample had a mean age of 16 years and was composed of females and males in quite similar proportions. Almost half of the participants were from central Italy. The mean length of competitive swimming was 8 years.

A total of 24 of competitive swimmers were diagnosed with COVID-19 in the previous season and for this reason they lost a mean amount of training days equal to 32.

The closure of indoor training facilities, due to pandemic prevention strategies to reduce SARS-CoV-2 transmission, caused an interruption in swimming training in about 18% of the participants (Table 2). The majority of these continued their training, mainly through home-based exercise. However, during the interruption, their weekly training time was substantially lower than they would regularly adopt (-8 median hours/week).

As for the comparison between athletes who maintained their training volume during the facility closures and those who did not, only the difference in regular weekly training volume was found to be significant between the two groups.

This variable was included in the logistic regression model, which also considered age and gender as covariates. Table 3 reports the results of the regression analysis.

A positive association was found between a regularly adopted weekly training volume higher than 12 h/week and that assumed during pandemic closure.

## 4. Discussion

The results of this study show that young competitive Italian swimmers were little affected by COVID-19 during the first phases of the COVID-19 pandemic. In fact, the proportion of athletes who reported SARS-CoV-2 infection in the sample examined was lower than that registered nationwide (cumulative incidence rate = 7.7% in August 2021), confirming that the swimming pool does not represent a high-risk place if adequate infection control measures are adopted [12,19]. This is in accordance with a study performed on a collegiate swim team in the USA, which reported an incidence value equal to 2% during the 2020/21 season [20].

Furthermore, it seems that the majority of the participants did not interrupt their training because of facility closure, since the government decrees issued in that period permitted pools used for the training of elite athletes to remain open [8,9,11].

Moreover, among those athletes who had to suspend their water activity, about 90% continued to exercise and approximately half maintained high levels of training, mainly at home. This is in line with other studies performed in other countries on different categories of athletes [21,22,23].

In the regression analysis, the weekly training volume normally adopted was found to be associated with achieving high levels of training even during the interruption of swimming due to pandemic closures, suggesting that habits adopted in the normal context of daily life played an important role in determining those adopted during the pandemic, as previously reported [22,24]. A study performed in 2020 by Harman et al. among outdoor endurance athletes showed that those of a higher athletic level exhibited greater lockdown resilience and coping strategies, perceived fewer barriers to training, and reported higher training volume during lockdown [25]. These findings suggest that resilience, which is fundamental to determine the individual’s adaptation to adverse conditions such as the unavailability of sports facilities, is strictly related to a high commitment to sports. At the same time, this underlines the importance of supporting those individuals who may not be able to respond to similar situations, such as amateur athletes or inactive individuals.

Some studies performed in other countries have shown that the pandemic influenced swimming performance in competitive athletes. In particular, a performance regression was registered during the 2020 French national championships, after eight weeks of lockdown without training, mainly among females, long-distance, and breaststroke swimmers [26]; the analysis of data points from Poland, Spain, Russia, Turkey, and Denmark championships from 2019 to 2020 showed improvements in symmetrical techniques, i.e., breaststroke and butterfly [27]; a performance analysis carried out on a collegiate USA swimming team between the 2020–2021 and 2019–2020 seasons highlighted significant reductions in swim training volume, with sprinters performing better and long-distance swimmers performing worse at the regional championships [20]; and a performance time deterioration in the 200, 300, and 400 m was found in Greek swimmers after an 11-week lockdown, while no changes were detected in 4 × 50 m and 50 m tests [28]. These findings suggest that the pandemic caused significant abstention from swimming and a reduction in training volume, leading to reduced aerobic fitness and impaired technical ability; when adopted, dryland training allowed the athletes to maintain strength and to preserve their sprint ability. Although this aspect is worthy of attention, we did not investigate the effects of training volume reduction on swimmers’ performance since it was not included in our goals. Further studies should be performed to analyze this and other possible effects of the COVID-19 pandemic in this population group.

This study has some limitations. First of all, it should be considered that swimmers were enrolled through an online invitation sent to their sport societies. Therefore, although the whole population of young competitive swimmers was considered for enrollment, only those teams and athletes who were keen to adhere to the study participated, probably determining a selection bias. Second, the cross-sectional design of the study and the method of questionnaire administration, although quick and inexpensive, could have caused the loss or inaccuracy of information, especially for those variables that concerned past events [29]. Furthermore, in order to control the length of the questionnaire we did not investigate some other sociodemographic, psychological, or training variables that may be related to the swimmers’ willingness to exercise in the changed conditions that they faced during the pandemic. Further studies should address these aspects in order to identify those determinants that should be enhanced to support people in maintaining or increasing their level of physical activity even during such emergencies.

The strength of this study is represented by the population examined. Although several studies performed in different countries have analyzed PA-related behaviors during the different phases of the COVID-19 pandemic, to the authors’ knowledge, no studies have examined the impact of this disease and related control measures on the category of young Italian competitive swimmers.

## 5. Conclusions

The COVID-19 pandemic has affected the practice of physical activity and sports, particularly watersports. The findings of this study show that young competitive Italian swimmers were little affected by COVID-19 and the related closure of facilities. Furthermore, the inability to access the pools did not represent an obstacle to training for the majority of those who were affected by mandatory closure, especially for those who normally follow high volumes of training. On the other hand, this draws attention to all of those non-elite athletes whose activity was particularly hindered by facility closures and were not able to replace swimming with other exercise. Further studies in this direction are needed to characterize the personal, environmental, and social resources that all individuals should have at their disposal to counteract the negative effects of similar measures adopted to control the spread of transmittable airborne diseases.

## Figures and Tables

**Table 1:** Characteristics of the participants and information on the effects of COVID-19 and related restriction measures on their training.

Variables	Total (n = 396)
Age	16.0 ± 3.2
(mean ± SD, median, IQR)	15, 14–17
Gender n (%)	
Female	202 (51.0)
Male	194 (49.0)
Geographical area n (%)	
North	117 (29.5)
Center	197 (49.8)
South	82 (20.7)
Training season n (%)	
Winter	197 (49.7)
Summer	199 (50.3)
Years of competitive swimming	
(mean ± SD)	8.0 ± 3.2
Number of training sessions/week	
(mean ± SD)	6.1 ± 1.3
Number of hours per training session	
(mean ± SD)	2.1 ± 0.3
Weekly training volume	12.6 ± 3.4
(mean ± SD, median, IQR)	12, 12–14
Cases of COVID-19 n (%)	24 (6.1)
Training days lost due to COVID-19 on n = 24	
(mean ± SD)	32.0 ± 15.3
Training interrupted due to pandemic closures n (%)	
no	326 (82.3)
yes	70 (17.7)
Training days lost due to pandemic closures	
(mean ± SD)	20.6 ± 28.2
Alternative training performed during pandemic closures n = 70 n (%)	
no	9 (12.9)
yes	61 (87.1)
Training places during pandemic closures n (%) on n = 61	
Physical activity indoor	35 (57.4)
Physical activity outdoor	7 (11.5)
Both	19 (31.1)
Number of training sessions/week during pandemic closures on n = 61	
(mean ± SD)	3.6 ± 1.8
Number of training hours per session during pandemic closures on n = 61	
(mean ± SD)	1.3 ± 0.5
Autonomous weekly training volume (hours x sessions) during pandemic closures on n = 61	4.9 ± 3.3
(mean ± SD, median, IQR)	4, 2–7.5

**Table 2:** Comparison between non- or less-training athletes and those who regularly exercised during pandemic closures.

	Training Volume ≤ 4 h/Week n = 35	Training Volume > 4 h/Week n = 26	*p*-Value
Age (mean ± SD)	16.8 ± 5.1	15.5 ± 1.7	0.122
Gender n (%)			0.554
Female	20 (57.1)	12 (46.1)	
Male	15 (42.9)	14 (53.9)	
Geographical area n (%)			0.844
North	9 (25.8)	6 (23.1)	
Center	20 (57.1)	14 (53.8)	
South	6 (17.1)	6 (23.1)	
Training season			0.996
Winter	20 (57.1)	14 (53.9)	
Summer	15 (42.9)	12 (46.1)	
Years of competitive swimming			0.476
(mean ± SD)	7.8 ± 3.4	9.2 ± 2.5	
Weekly training volume (total time per week)			
(mean ± SD)	10.9 ± 2.6	13.2 ± 4.5	0.020

Level of significance: *p*-value < 0.05.

**Table 3:** Results of the logistic regression analysis performed considering the maintenance of weekly training volume during pandemic facility closure as outcome.

Variable	OR (CI95%)	*p*-Value
Age		0.942
≤15 years	Reference	
>15 years	1.055 (0.246–4.521)	
Gender		0.570
Male	Reference	
Female	0.657 (0.154–2.796)	
Weekly training volume		0.012
≤12 h/week	Reference	
>12 h/week	9.433 (1.644–54.137)	

Level of significance: *p*-value < 0.05.

## Data Availability

The data are provided as tables and figures directly within the manuscript, and the raw data are available via email upon request to the corresponding author.
